# An MKT1 domain protein is dispensable for erythrocytic stages of *plasmodium falciparum*

**DOI:** 10.3389/fmicb.2026.1770301

**Published:** 2026-04-21

**Authors:** Baishali Chakraborty, Samantha M. McGee, Sudhir Kumar

**Affiliations:** Department of Biomedical Sciences, College of Veterinary Medicine, Iowa State University, Ames, IA, United States

**Keywords:** gametes, gametocytes, malaria, Mkt1, plasmodium

## Abstract

The life cycle of *Plasmodium falciparum* is complex, involving asexual and sexual reproduction in humans and mosquitoes, respectively. The parasite transmission from humans to mosquito vectors requires the formation of female and male gametes through gametogenesis. The MKT1 domain proteins are key molecules involved in posttranscriptional gene regulation and cellular differentiation in protozoans, plants, and animals, and the presence of an MKT1 ortholog suggested a possible role in stage differentiation in *Plasmodium*. Here, we report that *Pf*MKT1 was expressed in both asexual and sexual stages. Parasites with a *MKT1* gene deletion (*P. falciparum Pfmkt1¯*) proliferated asexually similar to wildtype NF54 parasites and differentiated into gametocytes forming mature male and female gametocytes. Further analysis showed that *P. falciparum Pfmkt1¯* gametocytes underwent gametogenesis to form male and female gametes and showed no apparent defect in flagellar gamete formation. This study identifies that MKT1-like protein is dispensable during asexual and sexual stage development.

## Introduction

The major causative agent of human malaria, *Plasmodium falciparum*, has a complex life cycle that involves a sexual cycle in female Anopheline mosquitoes and an asexual cycle in humans. Inside humans, parasites first proliferate asexually inside the hepatocytes and release hepatic merozoites. The merozoites initiate the asexual replication inside the erythrocytes, developing as ring, trophozoite, and schizont stages. Some of the asexually replicating parasites commit and differentiate into sexual forms known as gametocytes and develop as stage I-V. Gametocytes are terminal, non-replicating parasites that are taken up in an infectious blood meal by Anopheline mosquitoes. Inside the mosquito midgut, gametocytes rapidly differentiate into gametes with male gametocytes forming eight flagellar male gametes via three rapid rounds of mitosis, while a female gametocyte forms a single female gamete (Kumar et al., 2021). The fusion of male and female gametes leads to formation of zygote (Kumar et al., 2022) that transforms into an ookinete that develops into oocyst. The oocyst then undergoes asexual replication to produce sporozoites, which are released into a human host during onwards transmission (Venugopal et al., 2020; Chandley et al., 2022).

In eukaryotes, germ cell differentiation and gametophyte formation in plants are regulated by activation and/or repression of specific genes via genetic and epigenetic mechanisms by various regulatory factors (Ohinata et al., 2009; Nicholls et al., 2019; Hisanaga et al., 2019). Additionally, RNA-binding proteins play a critical role in regulating transcripts in plants (Ma et al., 2023) as well as protozoa (Alves and Goldenberg, 2016) during stress conditions. Furthermore, in *Plasmodium*, posttranslational mechanisms such as translational repression regulate gametogenesis and sporogony inside the mosquitoes (Cui et al., 2015). Like other eukaryotes (Protter and Parker, 2016), the translationally repressed transcripts are stored in punctate storage granules within the parasite cytoplasm. Several parasite proteins, including DOZI helicase (Mair et al., 2010; Min et al., 2024), CITH (homolog of worm CAR-I and fly Tailer Hitch) (Mair et al., 2006), ALBA proteins (Wang et al., 1993; Goyal et al., 2012), PUF proteins (Miao et al., 2013; Zhang et al., 2016), and the CAF1/CCR4/NOT complex (Hart et al., 2021; Hart et al., 2019), are involved in gene regulation through translational repression. Additionally, an RBP, MaCFET (Macrogamete-Contributed Factor Essential for Transmission) is a critical contributor to macrogamete fertility (Kumar et al., 2022).

The cytoplasmic poly (A)-binding proteins (PABPs) and PABP-binding protein 1 (Pbp1) are multifunctional RNA-binding proteins that regulate multiple aspects of mRNA translation and stability (Gray et al., 2015; Swisher and Parker, 2010). MKT1 (Maintenance of K2 Killer Toxin) is a part of the XPG/RAD2 nuclease family and forms a complex with Pbp1, that regulates stress responses (Tadauchi et al., 2004), and helps cells adapt to respiratory growth (Caballero et al., 2025). Overexpression of XPG/RAD2 proteins leads to genetic instability in both yeast and humans (Jimeno et al., 2017; Becker et al., 2018). Furthermore, in *Cryptococcus neoformans*, *CnMKT1* plays a key role during its sexual development and virulence by stabilizing mRNA in *Cryptococcus neoformans* (Son et al., 2019). In protozoan parasites such as *Trypanosoma brucei*, MKT1 is involved in maintaining mRNA stability by stabilizing the interactions between RNA-binding proteins (Singh et al., 2014).

In search of regulators of *P. falciparum* sexual development, we identified an MKT1 domain protein. *Pf*MKT1 expression is highest during stage V gametocyte stages. Interestingly, *PfMKT1* transcripts were downregulated in *Pfsrpk1¯* stage V gametocytes (Kumar et al., 2022). *Pfsrpk1¯* parasites show a complete block in gametogenesis (Kumar et al., 2022). This suggested a role for *Pf*MKT1 in sexual development. Here, we identify and characterize the role of *Pf*MKT1 in parasite asexual and sexual development.

## Results

### Identification of MKT1-like protein in *plasmodium falciparum*

**Figure 1:**
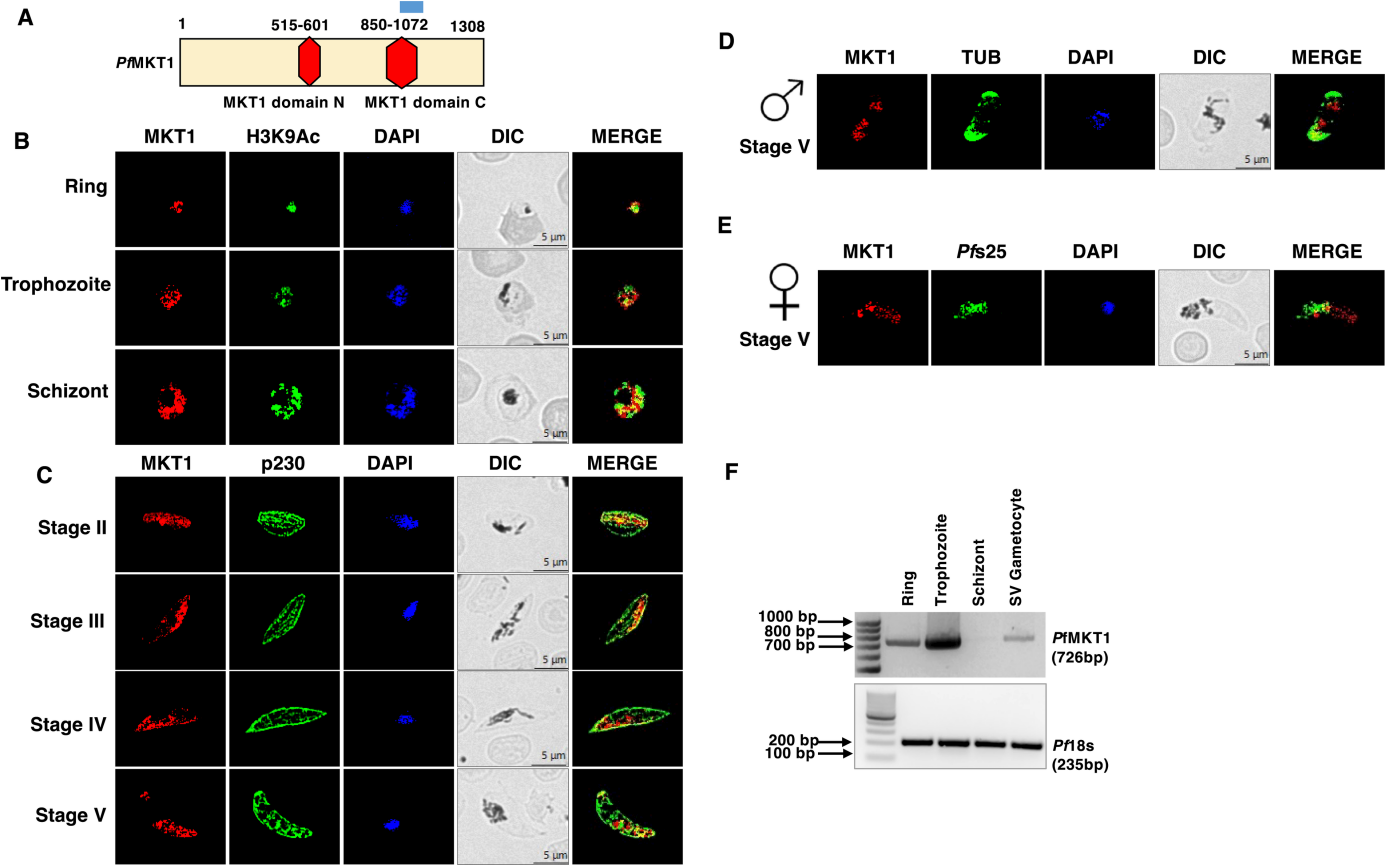
Expression and localization of *Pf*MKT1 in asexual and sexual parasite stages. **(A)** Domain architecture of *Pf*MKT1, which includes the predicted MKT1 domain N (Red) at residues 515 to 601 and the predicted MKT1 domain C (Red) at residues 850 to 1,072. The blue bar represents peptide region used for anti-MKT1 antisera generation. **(B)** IFAs were performed on the asexual (ring, trophozoite, schizont) stages using anti-MKT1 (red) and anti-H3K9Ac (green) antibodies. **(C)** IFAs were performed on the sexual (stage II-V gametocytes) stages using anti-MKT1 (Red) and anti-P230 (Green) antibodies. **(D)** IFAs were performed on the sexual stage V male gametocytes using anti-MKT1 (Red) and anti-Tubulin (green, male Marker) antibodies. **(E)** IFAs were done on the sexual stage V female gametocytes using anti-MKT1 (Red) and *Pf*s25 (green, female Marker) antibodies. The parasite DNA was stained with DAPI (blue). Scale bar = 5 μm. The representative images are shown from three experiments. Merge-merged image for red and green channels. Symbols for male and female gametocytes are shown on top of stage V gametocytes. DIC, differential interference contrast; DAPI, 4′,6-diamidino-2-phenylindole. **(F)** PCR was performed on the cDNA from ring, trophozoite, schizont and stage (SV) gametocyte stage parasites with *Pf*MKT1 ORF specific primer. *Pf*18s was used as loading control. Expected band sizes are indicated in bp.

The MKT1 ortholog in *Plasmodium falciparum* was identified through a homology-directed search of the *Plasmodium* genome on PlasmoDB that retrieved a gene with gene identifier PF3D7_1003700. *Pf*MKT1 contains a predicted MKT1 domain N and MKT1 domain C (Figure 1A). The presence of typical MKT1 N and C domains suggests a role for *Pf*MKT1 in post-transcriptional regulation. Interestingly, *PfMKT1* is present in human malaria parasites and in parasite species infecting nonhuman primates as well as in rodent malaria parasites. A broader similarity tree that included five human-infecting *Plasmodium* species, and a representative protozoan parasite *Trypanosoma* MKT1 revealed that *P. ovale* MKT1 shows comparatively higher similarity with *T. brucei*. Within the genus, *P. knowlesi* and *P. vivax* MKT1 proteins are closely related to each other. Likewise, *P. falciparum* and *P. malariae* MKT1 proteins also cluster together. (Supplementary Figure S1A). Strikingly, a further sequence alignment of *Plasmodium* MKT1 showed a high degree of divergence among species especially in MKT1 N and MKT1 C domains (Supplementary Figure S1B). A 3-D structure models generated using Alpha-Fold revealed prediction of MKT1 N and C domains with very high confidence (Supplementary Figure S1C). To determine its transcript level expression, we have performed semi quantitative RT PCR on Ring, trophozoite, schizont and mature gametocyte. Here, transcript of MKT1 (726 bp) was amplified using MKT1 specific primer. *Pf*18s was used as loading control. *Pf*MKT1 transcript is expressed in all the asexual stages and stage V mature gametocytes (Figure 1F). It is majorly expressed in trophozoite stage and faint expression was observed in schizonts.

### Analysis of *Pf*MKT1 expression during asexual and sexual stage development

The expression and localization of *Pf*MKT1 throughout the asexual and sexual stages of *P. falciparum* development was achieved using indirect immunofluorescence assays (IFA). Antisera were generated against the synthetic keyhole limpet hemocyanin (KLH)-conjugated peptide (DGKTYPENAYASIQNNNK), which was based on the C-terminal amino acid sequence of the *Pf*MKT1 protein and purified IgG was used for IFAs. The IFAs performed on thin culture smears demonstrated that *Pf*MKT1 is expressed during the asexual ring-, trophozoite-, and schizont stages (Figure 1B). Additionally, the IFAs on gametocyte stages showed that MKT1 is also expressed throughout the gametocytogenesis, including stage II-V (Figure 1C). Dual-label IFAs using antibodies against *α*-Tubulin and *Pf*s25 (markers for male and female gametocytes, respectively) and MKT1 revealed that *Pf*MKT1 is expressed in both male and female stage V gametocytes. (Figures 1D,E). *Pf*MKT1 appears to be predominantly localized in cytoplasm.

### *PfMKT1* could be deleted using CRISPR-Cas9-mediated transgenesis

To investigate the function of *PfMKT1* during asexual and sexual development, we successfully deleted *PfMKT1* using the CRISPR/Cas-9 strategy. DNA regions upstream and downstream of the *Pf*MKT1 locus were PCR-amplified and ligated through an overlapping linker, then cloned into the pFCL3 vector, which has previously been used for gene editing in *P. falciparum* (Goswami et al., 2020). Two 20-nucleotide guide sequences were also cloned individually generating two pFCL3_MKT1_KO plasmids. After transfection into wild-type (WT) *Pf*N54 parasites, homology-directed repair using the pFCL3 vector plasmid allowed the deletion of *Pf*MKT1 (Supplementary Figure S2A). The transgenic parasites were then drug-selected using 5 μM WR99210, PCR-genotyped, and subsequently cloned by limiting dilution. The *PfMKT1* locus deletion and clonality were confirmed by a set of genotyping PCRs (Supplementary Figures S2B, S2C). To further confirm the absence of the gene we have performed semi-quantitave RT PCR on cDNA of WT and *Pfmkt1¯* (clone F11 and H11) with two different MKT1 ORF-specific primers. Both of the ORF-specific primer pairs confirmed the absence of band in mutant denoting gene is completely knocked out (Supplementary Figures S2D, S2F). 18 s was used as loading control (Supplementary Figure S2E).

**Figure 2:**
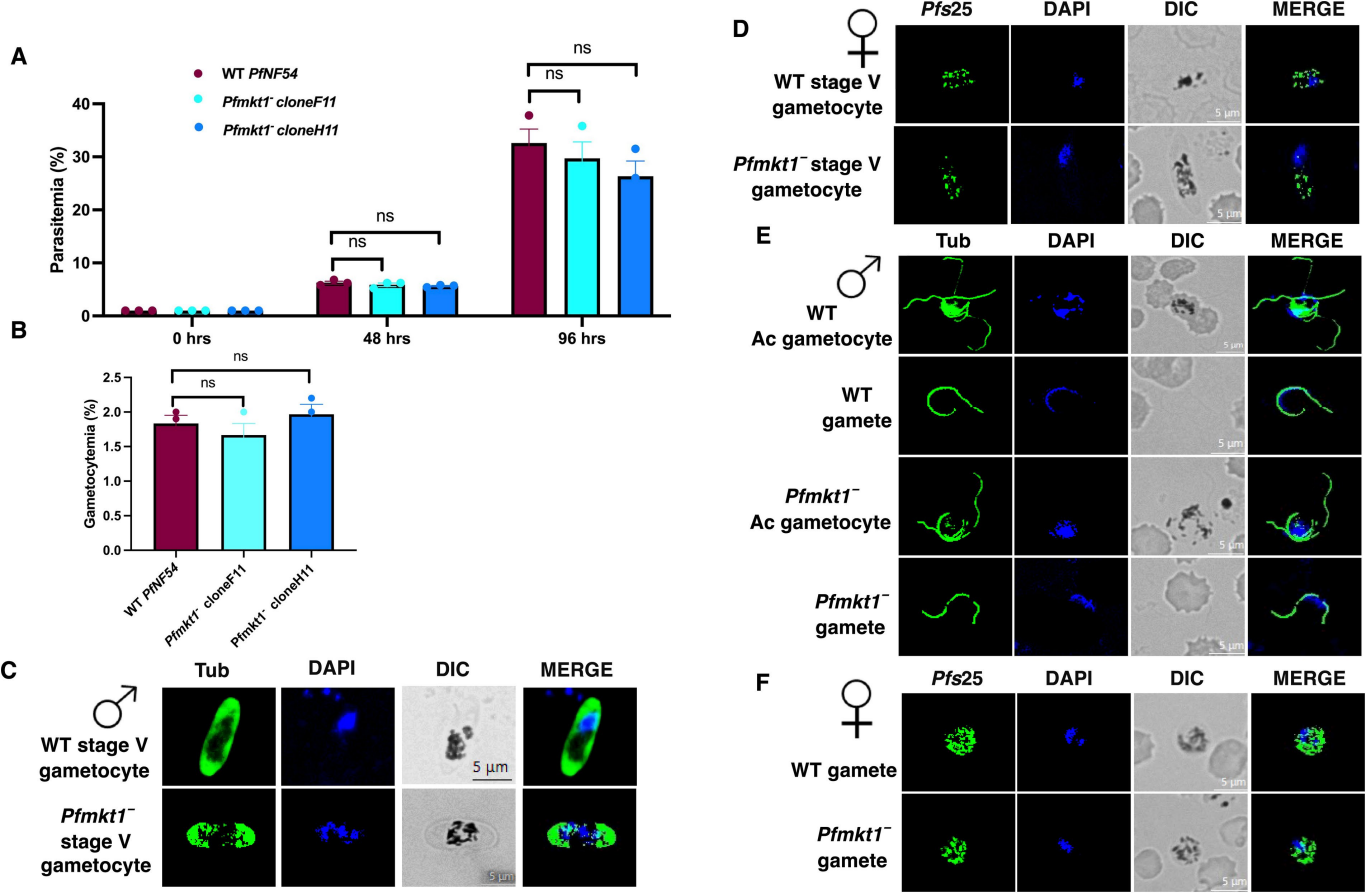
Characterizing gametocytogenesis and gametogenesis of *Pfmkt1¯* deficient parasites. **(A)** Growth assay performed on *Pfmkt1¯* parasites, using NF54 WT as a reference over the course of 96 h using Giemsa-stained thin culture smears. Data were averaged from three biological replicates and are presented as the mean ± standard error mean (SEM). ns; Non-significant. **(B)** Gametocytemia for *Pfmkt1¯* parasites, using NF54 WT as a reference. Data were averaged from three replicates and are presented as the mean ± standard error mean (SEM). ns; Non-significant. **(C)** IFAs were conducted on *Pfmkt1¯* and WT stage V male gametocytes using anti-Tubulin (green, male marker) antibodies. **(D)** IFAs were conducted on *Pfmkt1¯* and WT stage V female gametocytes using *Pfs*25 (green, female marker) antibodies. **(E)** IFAs were conducted on *Pfmkt1¯* and WT male gametes and activated male gametes using anti-Tubulin (green, male marker) antibodies. **(F)** IFAs were conducted on *Pfmkt1¯* and WT female gametes using anti-*Pf*s25 (green, female marker) antibodies. Scale bar = 5 μm. The representative images are shown from three experiments. Merge-merged image for red and green channels. Symbols for male and female gametocytes are shown on top of stage V gametocytes. DIC, differential interference contrast; DAPI, 4′,6-diamidino-2-phenylindole.

*Pfmkt1¯* parasites undergo normal asexual development. To assess the role of *Pf*MKT1 in asexual-stage development, growth rates were measured using growth assays on *Pfmkt1¯* parasite clones (F11 and H11) along with NF54 WT parasites as a control. Giemsa-stained smears were prepared every 48 h, which showed that the growth rate of the *Pfmkt1¯* parasite clones was similar to the WT references, indicating no significant defect in parasite asexual replication (Figure 2A**)**.

### *Pfmkt1¯* parasites undergo normal gametocytogenesis and gametogenesis

We next analyzed the ability of *Pfmkt1¯* parasites to generate gametocytes. For this, WT and *Pfmkt1¯* (clone F11 and H11) gametocytes were induced as described elsewhere (Tripathi et al., 2020). Percent gametocytemia was scored for all the cultures on day 15 of *in vitro* culture using Giemsa-stained thin culture smears. *Pfmkt1¯* parasites were able to develop into mature stage V gametocytes and had similar gametocytemia as the WT parasites (Figure 2B). Further IFAs performed on the *Pfmkt1¯* parasites using *α*-Tubulin and anti-*Pf*s25 antibodies revealed a normal development of female and male stage V gametocytes (Figure 2C and Figure 2D). We also tested the ability of *Pfmkt1¯* gametocytes to undergo gametogenesis. Day 15 gametocyte cultures for WT and *Pfmkt1¯* parasites were activated by addition of O^+^ human serum and a temperature drop from 37 °C to room temperature (RT). Further IFAs performed on the activated gametocytes and gametes using *α*-Tubulin and anti-*Pf*s25 antibodies revealed a normal development of female and male gametes for *Pfmkt1¯* parasites (Figure 2E and Figure 2F).

## Discussion

*Plasmodium* gametocytes are ingested by a mosquito vector during a blood meal, which then allows the gametocytes to undergo gametogenesis in the mosquito’s midgut. A male gametocyte produces eight flagellated male microgametes via three rounds of rapid mitosis. Due to the rapid nature of male gamete production, it is an error-prone process, leading to defects where the male gametes lack a nucleus and are therefore non-viable (Sinden et al., 1978; Mauer et al., 2023). Female gametocytes each form a single female macrogamete. This step represents a major bottleneck in the *Plasmodium* lifecycle, as very few gametocytes are ingested by the mosquito vector during natural transmission. The molecular mechanisms involved in regulating gametogenesis are largely unknown and parasite proteins including protein kinases such as CDPK1 (Bansal et al., 2018), CDPK4 (Kumar et al., 2021), SRPK1 (Kumar et al., 2022), MAP2 (Hitz et al., 2020), PKG (Baker et al., 2017), CRK5 (Kumar et al., 2022), a putative chromatin remodeler ARID (Kumar et al., 2022). Mitochondrial ATP synthesis is further shown to regulate gametogenesis in *P. falciparum* (Sparkes et al., 2024). This study identified a role for an MKT1-domain containing protein in parasite asexual and sexual stage development.

MKT1 proteins are a part of the XPG/RAD2 nuclease family and, in yeast, play a role in regulating stress responses and respiratory growth through post-transcriptional modifications (Caballero et al., 2025). MKT1 function in post-transcriptional gene regulation is also known in *Trypanosoma brucei* and *Cryptococcus neoformans*, where their respective MKT1 proteins function in stabilizing mRNA (Son et al., 2019; Singh et al., 2014). MKT1 was shown to be essential to the proper development of bloodstream and procyclic-form Trypanosomes (Singh et al., 2014). Additionally, MKT1 is essential for proper sexual reproduction in *Cryptococcus neoformans* and is required for pheromone gene expression. Due to MKT1’s role in gene regulation during cellular development, it was identified as a possible candidate for fertility regulation in *Plasmodium falciparum*.

*Plasmodium* genome encodes several RBPs with several of them showing high expression in gametocytes (Kumar et al., 2022; Miao et al., 2010; Shrestha et al., 2016). *Pf*MKT1 was recently identified in a proteomic study to interact with ring finger protein (*Pf*RNF1) and Male Development 3 (*Pf*MD3) (Farrukh et al., 2025). An MKT1 complex and its components have, however, not been identified in *Plasmodium.* It is thus possible that other RNA-binding proteins might bind to *Pf*MKT1 and regulate its function. Further, the phosphoproteomic data from PlasmoDB suggests that *Pf*MKT1 is phosphorylated at S357, S365, T385 an T389 in schizont stages. Furthermore, it is phosphorylated at S8 and S461 amino acid residues in the mature gametocytes (kumar lab, unpublished data), however, a protein kinase regulating this phosphorylation has not been identified. It is likely protein kinases that are highly expressed in mature gametocytes such as *Pf*SRPK1 (Kumar et al., 2022) and *Pf*CDPK4 (Kumar et al., 2022) phosphorylate MKT1 to regulate its RNA-binding function in the parasite.

This study shows that *Plasmodium* species encode a homolog of MKT1. Phylogenetic analysis showed that the *Plasmodium* MKT1 protein differs from the MKT1 ortholog of a different Apicomplexa parasite *T. brucei*. Interestingly*, P. ovale* MKT1 showed relatively higher similarity with *T. brucei* MKT1, than the other four human infective *Plasmodium* species. With *Plasmodium*, *P. knowlesi* and *P. vivax* MKT1 proteins are closely related to each other and so are *P. falciparum* and *P. malariae* MKT1 proteins. Further, multiple sequence alignment revealed dissimilarity in MKT1 N and C domains of different *Plasmodium* spp. This supports the concept of species-specific functional evolution. These observations also suggest differential selective pressures leading to homolog divergence among human-infecting *Plasmodium* species.

*Pf*MKT1 protein expression is the highest during Stage V gametocytes that suggested it may play a role in post-transcriptional regulation of genes associated with gametogenesis. Our results showed that MKT1 is not essential for parasite asexual development, despite *Pf*MKT1 being expressed throughout all asexual and sexual stages. Furthermore, *Pfmkt1¯* parasites showed normal exflagellation, indicating that microgamete formation was not affected upon *MKT1* gene deletion. The apparent redundant function of *Pf*MKT1 for asexual growth, gametocytogenesis, and male gamete formation suggests that its function in *P. falciparum* may diverge from its well-characterized roles in other eukaryotes. *Pf*MKT1 might have an important function inside humans and standard laboratory *in vitro* experiments may not be able to detect a function for *Pf*MKT1. However, *Pf*MKT1 might have a role in later stages of parasite development such oocyst development and sporogony that will be investigated in future studies. Interestingly, *Pf*MKT1 does not appear to be expressed during exoerythrocytic development (Zanghí et al., 2025). Given the high amino acid divergence within human-infective parasites, MKT1 might have an important function in other species and will be interesting to investigate in future studies.

## Materials and methods

### Reagents and antibodies

All the molecular biology reagents were purchased from Millipore Sigma (United States) unless stated otherwise. The primary antibodies and dilutions used for IFA were as follows: mouse *α*-tubulin antibody (1:400, Sigma Aldrich, Cat# T5168), mouse *Pf*s25 antibody (1:1, Bei resources, cat# MRA-28), mouse P230 antibody (1:350, Bei resources, MRA-878A), mouse H3K9Ac antibody Ref #MA5-31512 (1:200), and MKT1 purified IgG antibody (1:200). Anti-MKT1 antibody was raised by Biomatik Inc., USA and guidelines for antisera generation were followed. Pre-immune serum was used as a control to test the specificity of the antibodies. All Alexa Fluor conjugated secondary antibodies for IFAs (1:500) were purchased from Thermo Fisher Scientific, US.

### Parasite culture maintenance and generation of transgenics

The WT *PfNF54* and *Pfmkt1¯* parasites were cultured during asexual stages according to standard procedures. The parasites receive complete RPMI medium supplemented with either 0.5% AlbuMAX II (Thermo Scientific) medium or 10% (vol/vol) human serum every 24 h. The sexual gametocytes were generated using RBCs procured from BioChemed, United States, and O^+^ human serum from BioIVT, USA, using methods published elsewhere (Tripathi et al., 2020). The oligonucleotides that were used for the generation and analysis of *Pfmkt1¯* are shown in Supplementary Table S1. CRISPR-Cas9-based transgenesis methodology was utilized for the complete deletion of the *Pf*MKT1 gene (PlasmoDB identifier gene PF3D7_1003700). The gene deletion was then confirmed using genotyping PCRs shown in Supplementary Figure S2. The phenotypic characterization of *Pfmkt1^−^* parasites was conducted using two clones *Pfmkt1¯* F11 and H11.

### Measurement of parasite growth and gametocyte development

The asexual blood stage replication and growth were compared between the WT *PfNF54* and *Pfmkt1¯* parasites by setting up an initial ring stage parasitemia of 1% and culturing the synchronized parasites in 6-well plates. After 48 and 96 h, thin culture smears were prepared for the WT *PfNF54* and *Pfmkt1¯* parasites. Parasitemia was scored per 1,000 erythrocytes for Giemsa-staining preparation. Gametocyte formation was compared between the WT *Pf*NF54 and *Pfmkt1¯* parasites by culturing the parasites as stated above. On day 15 of *in vitro* culture, the parasites were removed, and Giemsa-stained thin blood culture smears were prepared. Parasitemia was scored per 1,000 erythrocytes.

### Indirect immunofluorescence assay and microscopy

For IFAs on gametocytes and exflagellating gametes, thin smears were prepared on Teflon coated slides and fixed with chilled methanol for 5 min (2 min for asexual stages). The slides were kept in humidity chambers to prevent them from drying out for each step. Fixed parasites were washed twice with PBS and permeabilized using 0.05% Saponin/PBS solution for 1 min. The slides were then washed twice with PBS for 5 min each and blocked with 3% bovine serum albumin (BSA)/PBS for 45 min. The primary antisera were diluted in 3% BSA/PBS and added to the slides, which were then incubated at 4 °C overnight. The antigens were visualized using anti-species antibodies. Keyence BZ-X 800 fluorescence microscope (KEYENCE America) was used to obtain images at 100 × 1.4-numerical aperture (NA) objective 90 (Nikon).

rt PCRs for transcriptional investigation by Semi-quantitative RT PCR. Total RNA was extracted from WT ring, trophozoite, schizont and stage V gametocytes using using TRI reagent (Sigma Aldrich #T3809) following manufacturer’s instructions. RNA concentration was determined by spectrophotometry, and RNA integrity was assessed by agarose gel electrophoresis. 1.5ug of total RNA was used as the template for cDNA synthesis using random hexamer primer and High-Capacity cDNA Reverse Transcription Kit (Applied Biosystems # 4368813). PCR was performed on the cDNAs with a primer inside *Pf*MKT1 open reading frame (ORF) along with 18 s loading control (Figure 1F).

### Statistical analysis

The data is expressed as the mean ± standard deviation (SD). Statistical differences were determined using unpaired two-tailed Student’s t test. In order to be considered statistically significant, the values of *p* < 0.05 were chosen. The significance of the data values was calculated using GraphPad Prism 9 and are shown in the figures as follows: ns, not significant (*p* > 0.05); *p < 0.05. *p* < 0.05; **, *p* < 0.01; ***, *p* < 0.001.

## Data Availability

The original contributions presented in the study are included in the article/Supplementary material, further inquiries can be directed to the corresponding author.

## Supplementary material

SUPPLEMENTARY FIGURE S1 Phylogenetic analysis of *Plasmodium* species MKT1. **(A)** Phylogenetic analysis of *Trypanosoma brucei* (*Tb*) and the five human-infecting *Plasmodium* species, which are *Plasmodium ovale* (*Po*), *Plasmodium falciparum* (*Pf*), *Plasmodium malariae* (*Pm*), *Plasmodium knowlesi* (*Pk*), and *Plasmodium vivax* (*Pv*). **(B)** Sequence alignment of the five human-infecting *Plasmodium* species. **(C)** Predicted three-dimensional structure of *Pf*MKT1 generated using Alpha Fold software. pLDDT is a per-residue measure of local confidence.

SUPPLEMENTARY FIGURE S2 Disruption of the *Pf*MKT1 locus. **(A)** *Pfmkt1¯* deficient parasites were generated using CRISPR-Cas9-mediated homology-directed repair (HDR). A DNA plasmid was created that contained genetic material that is homologous to the 5’ (upstream) and 3’(downstream) regions of the *MKT1* locus (5’ HR, 3’ HR). The DNA plasmid also contained the single guide RNA (sgRNA) and a template for Human-Dihydrofolate Reductase (hdhfr). **(B)** The deletion of *PfMKT1* was confirmed through genotyping PCR reactions. Oligonucleotides were designed to bind to loci peripheral to the *PfMKT1* open reading frame (ORF) (Primer 1, Primer 4), and intragenic loci (Primer 2, Primer 3). **(C)** The expected PCR sizes based on the primer combinations used in genotyping WT and *Pfmkt1¯* parasites. **(D)** Confirmation of gene deletion using PCR with two ORF specific primers (ORF1 and ORF2) on cDNA extracted from WT and *Pfmkt1¯* parasites. **(E)** PCR with *Pf*18s primers as loading control on cDNA extracted from WT and *Pfmkt1¯* parasites. **(F)** The expected PCR sizes based on the primer combinations used on WT and *Pfmkt1¯* parasites. **(G)** IFAs were performed on the asexual *Pfmkt1¯* schizont stage parasites using anti-MKT1 (red) and anti-Tub (green) antibodies. Scale bar = 5 μm. Images are shown from representative experiments. Merge-merged image for red and green channels. DIC, differential interference contrast; DAPI, 4′,6-diamidino-2-phenylindole.
