# Supplementary material for: An MKT1 domain protein is dispensable for erythrocytic stages of plasmodium falciparum
Source: Front Microbiol. 2026 Apr 21;17:1770301. doi: 10.3389/fmicb.2026.1770301 (PMC13139092; doi:10.3389/fmicb.2026.1770301)
Supplement: Supplementary file 1 [file Table_1.docx]

| **Oligonucleotides used for generation of *Pfmkt1¯* plasmids** | |
| --- | --- |
| **Oligo** | **Sequence (5’-3’)** |
| ***Pf*MKT1 5’Homo For** | TGCGGCCGCGCTGCGTGGAAATACATTTTTATTTATATTAAAT  AAATATATTTAATC |
| ***Pf*MKT1 5’Homo Rev** | CCAACCCGGGTATAGGCGCGCCTGGATACATATATCTATATAAA  GAAGAAACCCGACAAG |
| ***Pf*MKT1 3’Homo For** | AGGCGCGCCTATACCCGGGTTGGGACAAGAATTTTTATTAATTA  GTATTATAAGAATGC |
| **PfMKT1 3’Homo Rev** | TAAGTCGACCCTTTTGTGCGTGGAAATATTAGACCATATATAT |
| ***Pf*MKT1 Guide1 For** | TATTAAAAAAAAATGAGAGTTCGA |
| ***Pf*MKT1 Guide1 Rev** | AAACTCGAACTCTCATTTTTTTTT |
| ***Pf*MKT1 Guide3 For** | TATTGTAATAGGATTCACAAAACTC |
| ***Pf*MKT1 Guide3 Rev** | AAACGAGTTTTGTGAATCCTATTAC |
| **Oligonucleotides used for Genotyping of *Pfmkt1¯* parasites** | |
| ***Pf*MKT1_Geno5 For** | GAAATTACTTAAGGTTTATAATTTCTGAATTGGTAAAAG |
| ***Pf*MKT1 Geno5 Rev** | GCTGAAAATAATTTATGTCCTTTAGGTGTTATGCCG |
| ***Pf*MKT1 Geno3 For** | GATGAAGAATATTGTCAAACACCCATAAATGATGATTCAC |
| ***Pf*MKT1 3’Homo Rev** | TAAGTCGACCCTTTTGTGCGTGGAAATATTAGACCATATATAT |
| **Oligonucleotides used for Semi-quantitative RT PCR** | |
| ***Pf*MKT1 ORF1 For** | ACAGGACATAATGAAGATGGTGATCATGTTGATAATGC |
| ***Pf*MKT1 ORF1 Rev** | GGAATCTATAATAGGATGACTATAATCAATCCACTCACCC |
| ***Pf*MKT1 ORF2 For** | GCGACTATTCTAGTAATGATGTTGATAATTATG |
| ***Pf*MKT1 ORF2 Rev** | CCTTGTGTTCTACTTGATTTATACCAT |
| **18s For** | AATCTTGAACGAGGAATGCC |
| **18s Rev** | GGAAACCTTGTTACGACTTCTCC |

**Table S1. List of oligonucleotides used in this study.**
