# Supplementary figures and images for: An MKT1 domain protein is dispensable for erythrocytic stages of plasmodium falciparum
Source: Front Microbiol. 2026 Apr 21;17:1770301. doi: 10.3389/fmicb.2026.1770301 (PMC13139092; doi:10.3389/fmicb.2026.1770301)

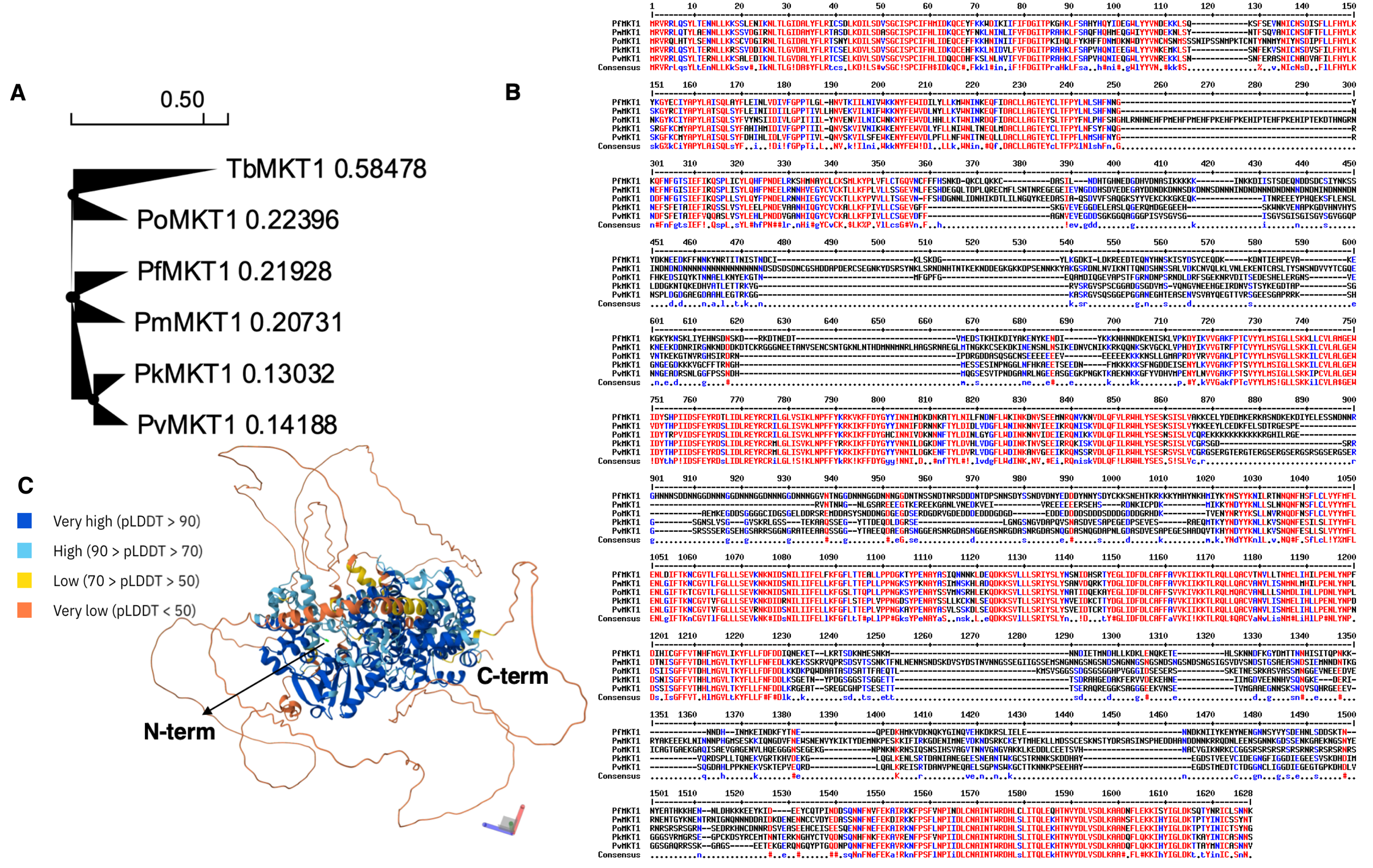

Supplement: SUPPLEMENTARY FIGURE S1 — Phylogenetic analysis of Plasmodium species MKT1. (A) Phylogenetic analysis of Trypanosoma brucei (Tb) and the five human-infecting Plasmodium species, which are Plasmodium ovale (Po), Plasmodium falciparum (Pf), Plasmodium malariae (Pm), Plasmodium knowlesi (Pk), and Plasmodium vivax (Pv). (B) Sequence alignment of the five human-infecting Plasmodium species. (C) Predicted three-dimensional structure of PfMKT1 generated using Alpha Fold software. pLDDT is a per-residue measure of local confidence. [file Image_1.tif]
